# The direct medical cost of type 2 diabetes mellitus in South Africa: a cost of illness study

**DOI:** 10.1080/16549716.2019.1636611

**Published:** 2019-07-08

**Authors:** Agnes Erzse, Nicholas Stacey, Lumbwe Chola, Aviva Tugendhaft, Melvyn Freeman, Karen Hofman

**Affiliations:** a SAMRC/Wits Centre for Health Economics and Decision Science - PRICELESS SA, School of Public Health, Faculty of Health Sciences, University of the Witwatersrand, Johannesburg, South Africa; b School of Human and Community Development, University of the Witwatersrand, Johannesburg, South Africa

**Keywords:** Cost of illness, type 2 diabetes mellitus, non-communicable diseases, South Africa, healthcare costs

## Abstract

**Background**: Type 2 diabetes mellitus (T2DM) is known to require continuous clinical care and management that consumes significant health-care resources. These costs are not well understood, particularly in low- and middle-income countries.

**Objective**: The aim of this study was to estimate the direct medical costs associated with T2DM in the South African public health sector and to project an estimate of the future direct costs of T2DM by 2030.

**Methods**: A cost of illness study was conducted to estimate the direct medical costs of T2DM in South Africa in 2018 and to make projections for potential costs in 2030. Costs were estimated for diagnosis and management of T2DM, and related complications. Analyses were implemented in Microsoft Excel, with sensitivity analysis conducted on particular parameters.

**Results**: In 2018, public sector costs of diagnosed T2DM patients were approximately ZAR 2.7 bn and ZAR 21.8 bn if both diagnosed and undiagnosed patients are considered. In real terms, the 2030 cost of all T2DM cases is estimated to be ZAR 35.1 bn. Approximately 51% of these estimated costs for 2030 are attributable to the management of T2DM, and 49% are attributable to complications.

**Conclusion**: T2DM imposes a significant financial burden on the public healthcare system in South Africa. Treatment of all prevalent cases would incur a cost equivalent to approximately 12% of the total national health budget in 2018. With rising prevalence, direct costs will grow if current care regimes are maintained and case-finding improved. Increased financial resources are necessary in order to deliver effective services to people with T2DM.

## Background

South Africa (SA) has a growing prevalence of diabetes mellitus (DM). In 2010, approximately 9% of men and 11.8% of women had diabetes, increasing almost twofold since 1980 when the NCD Risk Factor Collaboration reported a prevalence of 4.8% and 7.7% for men and women accordingly [1]. The latest (2012) age-standardised diabetes prevalence rate was 10.1% for the population aged 15 and above. Approximately 90% of this group suffers from type 2 diabetes mellitus (T2DM) [2] of which 60.1% is estimated to be unscreened and undiagnosed [3]. By 2015, complications from T2DM were the leading natural cause of death in women in South Africa, and the second largest cause for the entire population [4].

The emergence of diabetes has occurred alongside South Africa’s HIV/AIDS and tuberculosis (TB) co-epidemic, exacerbating the strain on a fragile public healthcare system which services over 80% of the population [5,6]. There is limited capacity to effectively deliver healthcare to those with non-communicable diseases (NCDs), while simultaneously providing treatment to populations with HIV/AIDS and other communicable diseases [7]. NCDs are ‘silent’ and often remain undiagnosed and untreated. Recent estimates show that as many as 80.6% of the population with DM (including both diagnosed and undiagnosed cases) in South Africa have an unmet need for care [3]. The 19.4% treatment coverage rate is far below the recommended 80% outlined by the World Health Organization (WHO) and the World Bank Group for countries committed to the achievement of Universal Health Coverage (UHC) [8]. The urgent need to address the NCD burden is also reinforced by several of the 2030 Sustainable Development Goals, most notably SDGs 3.4, 3.5, 3.8, and 3.9 [9].

Diabetes is a chronic disease, requiring continuous clinical care and management, which consumes significant health-care resources. The direct costs of managing T2DM and caring for diabetic sequelae such as renal failure, blindness and amputations include hospital and medication costs incurred by individuals, families, governments and private insurers. In 2015, the overall cost of diabetes in sub-Saharan Africa was USD 19.45 billion or 1.2% of gross domestic product (GDP). Over 55% of this was attributable to direct medical costs, with out-of-pocket payments likely to exceed 50% of the total health expenditure in many countries [10]. These costs have not been enumerated in South Africa. Cost estimates have focused on the broader sub-Saharan African region [10–12], and the two forms of diabetes (type 1 and 2) are often conflated. Furthermore, little distinction is made between diagnosed and undiagnosed cases [10,11,13]. The development or improvement of cost-effective interventions for the management and treatment of diabetes depends on nuanced estimates on specific categories of treatment and complication costs. It is thus of vital importance to understand the resource implications of the burden of diabetes in South Africa in the context of proposals for National Health Insurance (NHI).

To address this gap, the aim of this study is to estimate 2018 direct medical costs associated with T2DM in the South African public health sector, and project future such direct costs for 2030.

## Methods

The study adopted a ‘bottom-up’ (person-based) approach to estimating the direct cost of diagnosis and control of T2DM and of its complications in public facilities, using a standard cost of illness (COI) approach [14]. Type 2 diabetes mellitus is defined in this study as a chronic disease characterised by hyperglycemia and dyslipidemia due to underlying insulin resistance. Information on the study is described in Table 1.

**Table 1.** Overview of the study approach.

Study objective	To estimate the direct medical costs associated with T2DM in the South African public health sector
Target audience	Governments to prioritise T2DM investments
Grouping of direct costs	(1) T2DM control and (2) T2DM-related complications: renal disease, diabetic eye diseases, diabetic foot disorder, diabetic heart disease
Time frame	One year
Costing approach	Bottom-up approach
Calculating the total annual cost	TC = UC x Pop Total cost (TC) estimates for T2DM were calculated by multiplying the unit costs (UC) by the target population (Pop)

Data were collected on key parameters within four data categories: population estimates, epidemiological parameters, treatment coverage, and direct medical cost (Table 2). T2DM-related chronic complications included chronic renal disease, diabetic eye diseases (cataracts, retinopathy), diabetic foot disorder (amputations), diabetic heart disease (stroke, ischaemic heart disease).

**Table 2.** Data categories and sources.

Data category	Parameter	Source
Population estimates	Population (15+) (n)	Statistics South Africa mid-year population estimates, 2018
Epidemiology parameters	Disease burden Medicine and technology need and usage Hospitalisation Outpatient visits	Literature review
Treatment coverage	Screened, diagnosed, treated, controlled cases	South African National Health and Nutrition Examination Survey (SANHANES-1, 2011–2012)
	Private/public sector divide	Council for Medical Schemes
Direct medical costs	Medication	South African National Health Laboratory Service
	Examinations and investigations	Uniform Patient Fee Schedule 2018
	Health workers wages	Department of Public Service and Administration

### Prevalence data and population estimates

Prevalence data were sourced from the first comprehensive national survey on NCDs, the South African National Health and Nutrition Examination Survey (SANHANES-1 (2011–2012)) [15]. To estimate the population with diabetes, prevalence data was applied to the adult population size (15+ age group) [16]. Year-specific population estimates were obtained from Statistics South Africa mid-year population projections. The prevalence of type 1 and type 2 diabetes was based on a split of 10% and 90%, respectively, of overall diabetes prevalence [2].

The number of patients treated at public health facilities was based on figures from the Council for Medical Schemes, which show that 84% of the population is uninsured [6]. Current treatment coverage was obtained from SANHANES-1 (2011–2012) [15].

To determine projected costs for 2030, South African population estimates were obtained from Spectrum population prospects data [17]. Projected T2DM prevalence was based on the mean annual increment of 55,000 diabetes cases, an estimate generated by Guariguata et al. [18] based on the latest high-quality data available [19,20], using the widely cited International Diabetes Federation`s methodology for generating country-level estimates of diabetes prevalence [21].

The proportion of complications for T2DM patients was based on a targeted literature search [11,22]. The concept of complications is often used interchangeably with comorbidities in public health research. For the purpose of this study, we define a complication as a condition that appears after diagnosis of the index case, in this case, T2DM.

### Costing

Cost components in this research were identified using national guidelines including the Society for Endocrinology, Metabolism and Diabetes of South Africa (SEMDSA) guidelines for the Management of Type 2 Diabetes 2017 [23], and the Updated Management of Type 2 Diabetes in Adults at Primary Care Level [24]. Cost data were collected and disaggregated by treatment and management of T2DM and that of complications. Direct cost related to the treatment and management of T2DM include the total annual cost of medication (insulin and oral drugs) and equipment (syringes and glucose meters); the total annual cost of diabetes-related test (HBA1c test, lipid profile, urea and electrolytes, cholesterol, microalbumin, ECG and retinal screening); and cost associated with the diagnosis and treatment of T2DM (including outpatient consultation and hospitalisation costs).

The valuation of examinations and investigations was based on cost information from the Uniform Patient Fee Schedule (UPFS) 2018 of the National Department of Health (NDoH) that provides cost by the different treatment parameters [25]. Health workers wages were obtained from the Department of Public Service and Administration database [26], and medication costs were extracted from the South African National Health Laboratory Service Report 2018 [27]. Where cost information for the public sector was not available, the National Health Reference Price List [28] was used. This is the latest (2006) legal tariff document for the public sector, assuming public sector costs to be 70% of private sector costs.

Annual cost of diabetes-related tests was based on clinical recommendations outlined in the SEMDSA 2017 that patients with DM annually require and receive one HBA1c test, one lipid profile test, one proteinuria test, one electrocardiogram, and one retinal screening. Outpatient and hospitalisation cost was estimated by applying resources used in the diagnosis and treatment of T2DM complications to appropriate unit costs. For costs resulting from patient visits, it was assumed that all T2DM cases make four visits per year to an outpatient clinic [29] and that 5% of T2DM patients would require one hospitalisation per year [30]. UPFS also includes facility fees per consultation and procedure. This was included for treatment and management activities occurring within facilities. Human resource cost estimates were based on general medical practitioners at Level 1 facilities who were diagnosing and managing T2DM patients. Complications were assumed to be treated and managed at Level 3 facilities by a specialist medical practitioner. Compensation of health-care personnel was based on a capitation model where health service providers are paid a fixed fee for the care of T2DM patients. The methodology used in the capitation model is described in Bourdon et al. [31].

Medication usage was obtained from the Centre for Diabetes and Endocrinology [32] and literature. The total cost of medication and equipment was based on the assumption that approximately 49% of T2DM cases use only oral medication, 9% use only insulin and 40% use a combination of insulin and oral drugs [33]. The quantity of glucose strips and lancets needed for blood spot glucose tests was based on an assumed monthly usage of a hundred component units per patient as well as syringes and needles for those who are insulin users.

Using the Consumer Price Index [34], costs have been adjusted to the same base-year South African Rand (2018) for comparison. Costs for 2030 were estimated based on the growth in the prevalence of T2DM, with the cost inflation over the time period being disregarded. Cost estimates are shown in 2018 South African Rand (ZAR) as well as in US dollars (USD).

Annual direct cost was quantified for two scenarios. First, to show how much is currently being spent on T2DM in the public sector and second, to estimate the additional cost if the UHC target is achieved by 2030.

### Costs of T2DM complications

The share of T2DM patients who develop diabetic eye, foot and heart disease (summarised in Table 3) was based on previous research [22]. Using these estimates, we applied incidence rates of people with the condition relative to the size of the population in our base year (2018).

**Table 3.** Estimated annual incidence of T2DM-related conditions.

Indicators	Total number of new cases per year
T2DM-related blindness	12 614
T2DM-related amputation	3 311
T2DM-related stroke	11 037
T2DM-related ischemic heart disease	8 672

Cost of diabetic eye disease was calculated by adding the cost of retinopathy screening, laser treatment for retinopathy, and treatment of cataracts, and multiplying by the number of patients in need of care. Diabetic foot care costs were expressed as the cost of amputation and the cost of below-knee prosthesis, multiplied by the number of patients.

Targeted literature searches indicate that approximately 40% of people with T2DM will develop kidney disease [24], among whom the annual incidence rate for progressing to dialysis is 113 per 100 000 [35]. The proportion of T2DM patients on haemodialysis (HD) is 71.8% of the total number of people on renal replacement therapy and 13.5% of those on peritoneal dialysis (PD) [36]. Further, 14.7% of patients undergo transplantation. Total cost of renal disease was obtained as follows: $TCRD=\left(CI+CH+CPD+CT\right)xNN$, where CI is the total cost of investigations for T2DM patients in need of renal replacement therapy; CH is the annual cost of haemodialysis; CPD is the annual cost of peritoneal dialysis; CT is the cost of kidney transplant; and NN is the number of patients in need each year. To estimate costs related to HD the following formula was used: Cost of HD in a year=2 sessions per week x 52 weeks per year x HD cost per session.

### Sensitivity analysis

The research was based on a range of assumptions and sources, with some resulting uncertainty in the estimated annual cost of T2DM. Uncertainty was managed by performing sensitivity analysis assuming ±20% change in the costs of medications, and the management of renal disease to examine how sensitive the estimates are to fluctuations in cost. A mean 20-point change was chosen in line with the South African Medicine Price Registry`s [37] historical trends in annual fluctuation in drug prices (range of 15–25%).

*Microsoft Excel*® was utilised for data capture and analysis.

## Results

T2DM prevalence is estimated to be over 3.1 million with 240 000 patients diagnosed and treated in the public sector. It is estimated that 4.5 million South Africans in the public sector will have T2DM by 2030.

The total direct cost (Table 4) attributable to the 240 thousand T2DM patients who were diagnosed, treated and controlled in 2018 was over ZAR 2.7 bn (USD 198 million). Direct costs were broken down by treatment and management of T2DM and that of complications. Over half of the cost (ZAR 1.4 bn, USD 102 million) was attributable to treating and managing T2DM, while the cost of complications was estimated at ZAR 1.3 bn (USD 96 million).

**Table 4.** Estimated direct medical health-care costs of T2DM in 2018, in thousands of South African Rand and US Dollars.

			Total annual cost		
	Diagnosed T2DM cases (n = 240 447)			Diagnosed and undiagnosed T2DM cases (n = 3 106 321)	
	(ZAR)	(USD)		(ZAR)	(USD)
Medication and equipment					
Total annual cost of insulin	828 047	59 619		10 697 464	770 217
Total annual cost of syringes and needles	1 138	81		14 705	1 058
Total annual cost of glucose strips and lancets	4 635	333		59 885	4 311
Total annual cost of glucose meter and batteries	4 320	311		55 816	4 018
Total cost of oral drugs	96 739	6 965		1 249 763	89 983
Patient visit					
Total cost of OPD consultation	47 608	3 427		615 051	44 283
Total cost of hospitalisation	99 437	7 159		1 284 619	92 492
Total cost of diabetes-related tests	338 340	24 360		4 370 987	314 711
Complications					
Laser treatment for retinopathy and follow-up outpatient consultation	20 888	1 503		20 888	1 503
Treatment of cataracts	29 232	2 104		29 232	2 104
Amputation*	83 285	5 996		83 285	5 996
Stroke	383 226	27 592		383 226	27 592
Ischaemic heart disease	187 296	13 485		187 296	13 485
Renal imagining	182 505	13 140		2 357 772	169 759
Renal biopsy (per kidney)	429	30		429	30
Total annual cost of Haemodialysis	369 328	26 591		369 328	26 591
Total annual cost of Peritoneal Dialysis	61 065	4 396		61 065	4 396
Total cost of kidney transplant	18 129	1 305		18 129	1 305
Total cost of investigations	5 244	377		5 244	377
Total cost excluding complications	1 420 268	102 259		18 348 294	1 321 077
Total cost of complications	1 340 632	96 525		3 515 898	253 144
GRAND TOTAL	2 760 900	198 784		21 864 193	1 574 221

*including hospitalisation; below knee prosthesis.

The additional cost of treating all of those in need, both diagnosed and undiagnosed cases, would be ZAR 19.1 bn. This represents an 8.9-fold increase in expenditure, accounting for approximately 12% of the national health budget.

When broken down by resource category (Table 5), pharmacologic costs accounted for 33% of total expenditure. This included insulin and oral antidiabetic drugs; medication costs associated with the treatment of co-morbidities were included in the complications category. A further 4% was attributable to hospitalisation; 12% consisted of diabetes-related tests (HBA1c, lipid profile, urea & electrolytes, cholesterol, microalbumin, ECG and retinal screening); and 2% consisted of outpatient consultations. Compensation of health-care professionals did not have its own category due to the capitation model used in the methodology where health service providers are compensated on a fixed fee basis for the care of T2DM patients.

**Table 5.** Total direct cost attributable to diagnosed T2DM in 2018, in thousands of South African Rand and US Dollars.

Resource category	Cost in thousands of South African Rand and US Dollars		% of annual total direct cost	% of South Africa`s annual healthcare budget
	**(ZAR)**	**(USD)**		
Medication	924 787	66 584	33	0.52
Investigations	338 340	24 360	12	0.19
Hospitalisation	99 437	7 159	4	0.06
OPD consultations	476 086	3 427	2	0.03
Complications	1 340 623	96 525	49	0.75
TOTAL DIRECT COST	2 760 900	198 784	100	1.55

The management of retinopathy and renal disease was the two main cost drivers of the total expenditure, and together are responsible for over two-thirds of the costs related to complications. A single haemodialysis session, for example, costs ZAR 3 147 meaning that the annual treatment of renal failure for a single patient attending two sessions per week is ZAR 503 399.

The sensitivity analysis (Table 6) shows that direct public healthcare cost estimates are moderately sensitive to a 20% change in costs related to medication and the treatment and management of the renal disease.

**Table 6.** Sensitivity analysis for annual T2DM costs in hundred thousands of South African Rand and US Dollars.

Variable		Medicine		
Treatment and management of renal disease		−20%	0%	20%
	−20%	2 564 ZAR (174 USD)	2 633 ZAR (179 USD)	2 818 ZAR (191 USD)
	0%	2 575 ZAR (175 USD)	2 760 ZAR (187 USD)	2 945 ZAR (200 USD)
	20%	2 703 ZAR (183 USD)	2 888 ZAR (196 USD)	3 247 ZAR (220 USD)

Results of the projection for 2030 show a significant increase in the costs of treating all T2DM and related complications. Accounting for an estimated incidence rate of 55,000 new diabetes cases, the burden of T2DM is projected to cost the South African public healthcare system over ZAR 35.1 bn (USD 2.5 bn) by 2030.

## Discussion

We estimated the total annual direct cost to the public health sector, attributable to the 240 thousand T2DM patients who are diagnosed, treated and controlled, to be approximately ZAR 2.7 bn. This accounted for about 1.55% of South Africa`s health-care budget in 2018. If treatment coverage is increased for all T2DM patients who are in need of care there would be an 8.9-fold increase in expenditure, accounting for 12% of the health-care budget. This amount forecasts the financial needs to treat and manage T2DM as the country moves toward UCH through NHI and represents the opportunity cost of resources used for treatment.

There is an emerging body of literature that estimates the magnitude of the cost of T2DM in both high- and low-income countries to which our study relates [36,38]. Our results are consistent with other studies in the sub-Saharan Africa region where medication represents a significant amount of the direct costs [11,36]. Studies projecting costs of diabetes based on the future development of diabetes prevalence over a certain period of time also showed increasing estimates in diabetes-attributable health-care costs [38] with some variation in their scope. A possible explanation for the variation might relate to methodological disparities across studies. For Australia, researchers extrapolated the total cost of diabetes from the year 2000 to 2051, estimating that the cost of diabetes would be 2.5 to 3.4 times higher in 2051 [39]. In this case, assumptions underlying the methodology included a 1:1 ratio of diagnosed and undiagnosed cases and that the cost of undiagnosed cases was 50% of that of known cases, leading to lower results. An even lower increase in the total direct medical costs (1 · 8 fold) was reported in China, where researchers only included diagnosed cases when estimating costs attributable to the expected growth in T2DM prevalence [40].

The scope of this study was limited to estimating the public health expenditure on chronic complications; however, costs related to comorbidities – that may develop in addition to T2DM – cannot be overlooked. Recent SA research investigating diabetes comorbidities shows that 73% of people with DM had at least one additional chronic illness [12]. For example, the costs of antihypertensive therapy for the estimated 72% of patients with T2DM suffering from high blood pressure would increase the total direct cost to the public health sector by 16.7% annually [12]. Against the background of the high cost of NCD comorbidity, the health sector also faces the simultaneous economic burden of comorbidities with other chronic infections. In South Africa in particular, T2DM is associated with a two to three-fold higher risk of developing active TB disease and is responsible for increased TB treatment failure, relapse, and death [41]. In light of the rising prevalence of T2DM and the prevailing high burden of TB in South Africa, the question arises as to how best to manage comorbidities in a setting with already overburdened public health services.

Furthermore, while indirect costs were not the primary interest of the study, a recent systematic review on the cost of T2DM in LMICs indicates that annual indirect cost per T2DM patient could range between USD 1.92 to USD 73.4 (ZAR 27.1 and ZAR 1059.46) [42]. If these estimates are extrapolated to the South Africa specific prevalence estimates, it would lead to a total amount of ZAR 83 million to ZAR 3.2 bn per year.

Several limitations must be considered. First, the analysis applied a prevalence-based approach that included a cross-section of T2DM cases. This reflects costs at varying stages of the disease, including cases that may not be amenable to intervention. Therefore, assumptions relating to hospital visits and diagnostic tests might lead to overestimation of direct costs, as not all T2DM patients will be able to make contact with health-care providers within the defined period (one year). Furthermore, the prevalence-based approach is static and not dynamic, which means forecasting costs in the future are done by a simple assumption of increased prevalence rather than modelling of disease progression. Second, varying levels of access and adherence to medication and T2DM services were not accounted for due to data constraints. On the one hand, this might lead to overestimates of some unit costs (medication and consumable); on the other hand, it provides an underestimation of indirect costs by discounting expenditure resulting from investments into healthcare infrastructure to make medication and services available to those in need. Third, cost components did not represent an exhaustive list. There are other health-care professionals (podiatrist, physiotherapist), and services such as education courses targeted at people with diabetes, or nursing home stays for which costs were not obtained, and thus the total direct costs in this analysis are potentially an underestimate.

## Implications

Our findings indicate the cost of the treatment and management of T2DM in the public sector are significant, even with a high prevalence of undiagnosed cases. The South African NDoH has identified a set of effective policy interventions for the prevention and control of NCDs [43]; however, the need for more effective implementation of preventive primary and secondary measures is urgent. The implementation of preventive and care services for diabetes falls short on the desired goals in Africa, with more than half of the cases not meeting recommended targets to prevent expensive to treat complications [44]. Diabetes prevention guidance issued by the SEMDSA favours intensive lifestyle programmes (which can be difficult to implement) and pharmacological interventions [23]. Primary prevention through improved diagnoses, targeted screening of higher-risk individuals, and pharmacological management of type 2 diabetes must be a priority to reduce the incidence of type 2 diabetes and its associated complications in South Africa [45].

Further, in light of the prominence of medication costs being a significant driver of T2DM costs, attention must be paid to improved policies, planning and prioritisation for diabetes, with particular attention to the cost of medications. If there is increased screening and coverage is expanded to all un-diagnosed cases, spending on medications will significantly increase. Ideally, with appropriate health technology assessment processes in place, it would be possible to inform, constrain, and increase efficiencies in the procuring of additional medications.

At present, cost accounting in the public sector is not undertaken with reference to disease code to allow for tracking expenditure by disease. Beyond the particular case of T2DM we consider, it would be valuable for the public sector heading towards NHI to more accurately track expenditure and resources used across disease areas. Health expenditure data have been successfully applied in other low- and middle-income countries (LMICs) [46] to improve resource allocation and to better meet the objectives of the health system.

To understand the economic rationale for investments that reduce T2DM, future research should focus on the lifetime economic burden, including both direct and indirect costs of T2DM. It will be important to show short- and long-term indirect costs to society in terms of loss of patients’ incomes, the cost to employers in terms of loss of productivity, and the costs resulting from the provision of other social services such as disability grants. For example, in the case of patients with T2DM-related blindness alone, is approximately ZAR 12 120 per year per disabled person [47]. Future studies should analyse the return on investment and apply a ‘holistic’ approach to demonstrate social, economic and environmental value created by an intervention or policy. This would enable the incorporation of ethical and equity considerations to priority setting processes.

## Conclusion

This is the first study to look at the direct cost of treating and managing patients with T2DM and related complications in the SA public sector. As South Africa aims to fully implement NHI by 2025 and given the existing strain on the health-care sector alongside limited resources, setting priorities and service packages that address equity is crucial. Improved understanding of the annual cost of diabetes is critical because of the inevitable trade-offs that will need to be considered. Robust cost data on the burden of diabetes on health-care budgets, on the individual and families, and on the country’s GDP are essential. Cost estimates are indispensable and form the basis of prioritising interventions and simulation studies. Cost savings from prevention interventions can also then be estimated.

## Paper context

This study reports on public health expenditure on DM in South Africa. Not only does this analysis focus on T2DM specifically but accounts for undiagnosed cases and complications. The study addresses current gaps in knowledge regarding direct medical costs. This information is essential for further research and economic analyses that could provide the basis for return on investments and a better understanding of how to allocate resources more equitably.
